# Kaempferol induces hepatocellular carcinoma cell death via endoplasmic reticulum stress-CHOP-autophagy signaling pathway

**DOI:** 10.18632/oncotarget.19200

**Published:** 2017-07-12

**Authors:** Haiqing Guo, Wei Lin, Xiangying Zhang, Xiaohui Zhang, Zhongjie Hu, Liying Li, Zhongping Duan, Jing Zhang, Feng Ren

**Affiliations:** ^1^ Department of Hepatitis C and Drug-Induced Liver Disease, Beijing Youan Hospital, Capital Medical University, Beijing 100069, China; ^2^ Beijing Institute of Hepatology, Capital Medical University, Beijing 100069, China; ^3^ Department of Cell Biology, Municipal Laboratory for Liver Protection and Regulation of Regeneration, Capital Medical University, Beijing 100069, China; ^4^ Artificial Liver Center, Beijing Youan Hospital, Capital Medical University, Beijing 100069, China

**Keywords:** kaempferol, hepatocellular carcinoma cell, endoplasmic reticulum stress, C/EBP homologous protein, autophagy

## Abstract

Kaempferol is a flavonoid compound that has gained widespread attention due to its antitumor functions. However, the underlying mechanisms are still not clear. The present study investigated the effect of kaempferol on hepatocellular carcinoma and its underlying mechanisms. Kaempferol induced autophagy in a concentration- and time-dependent manner in HepG2 or Huh7 cells, which was evidenced by the significant increase of autophagy-related genes. Inhibition of autophagy pathway, through 3-methyladenine or Atg7 siRNA, strongly diminished kaempferol-induced apoptosis. We further hypothesized that kaempferol can induce autophagy via endoplasmic reticulum (ER) stress pathway. Indeed, blocking ER stress by 4-phenyl butyric acid (4-PBA) or knockdown of CCAAT/enhancer-binding protein homologous protein (CHOP) with siRNA alleviated kaempferol-induced HepG2 or Huh7 cells autophagy; while transfection with plasmid overexpressing CHOP reversed the effect of 4-PBA on kaempferol-induced autophagy. Our results demonstrated that kaempferol induced hepatocarcinoma cell death via ER stress and CHOP-autophagy signaling pathway; kaempferol may be used as a potential chemopreventive agent for patients with hepatocellular carcinoma.

## INTRODUCTION

**Figure 1:**
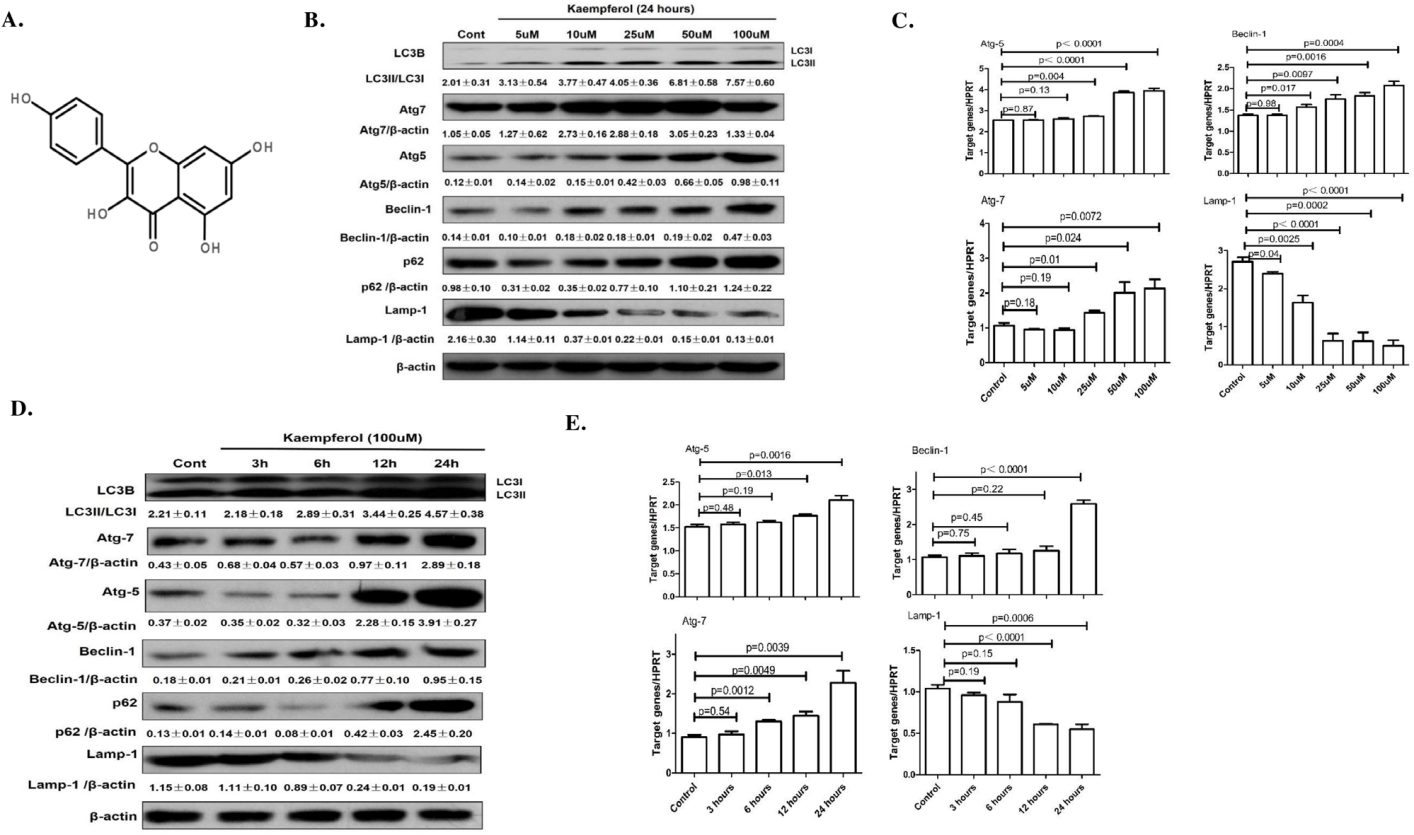
Dose- and time-dependent effects of kaempferol on protein and mRNA levels of autophagy-related genes **(A)** Chemical structure of kaempferol. **(B)** For dose-dependent effects, HepG2 cells were treated in the presence or absence of different concentrations of kaempferol for 24 h. The protein expression levels of Atg5, Atg7, Beclin1 and LC3B were measured by western blot. Data are expressed as mean ± SD of at least three independent experiments. **(C)** For dose-dependent effects, HepG2 cells were treated in the presence or absence of different concentrations of kaempferol for 24 h. The mRNA expression levels of Atg5, Atg7, Beclin1 and Lamp1 were measured by qRT-PCR. Data are expressed as mean ± SD of at least three independent experiments. **(D)** For time-dependent effects, HepG2 cells were exposed to 100 μM kaempferol for different time periods. The protein expression levels of Atg5, Atg7, Beclin1 and LC3B were measured by western blot. Data are expressed as the mean ± SD of at least three independent experiments. **(E)** For time-dependent effects, HepG2 cells were exposed to 100 μM kaempferol for different time periods. The mRNA expression levels of Atg5, Atg7, Beclin1 and Lamp1 were measured by qRT-PCR. Data are expressed as mean ± SD of at least three independent experiments.

Kaempferol (Figure 1A) is a flavonoid compound that is found in a variety of vegetables and fruits [1–3]. Kaempferol has long been used in traditional medicine to treat several diseases and has attracted widespread attention due to its pleiotropic biological functions, including antioxidant [4–5], anti-inflammatory [6–7] and antitumor [8–9] properties. The studies have demonstrated that kaempferol can invoke a number of different mechanisms in the regulation of cancer cells. For instance, kaempferol inhibited cell growth and migration of pancreatic cancer through the blockade of epidermal growth factor receptor (EGFR) -related pathway *in vitro* [10]. Moreover, kaempferol increased the effects of radiation on tumor cell killing *in vitro* and *in vivo* through inhibition of AKT/PI3K and ERK pathways and activation of mitochondrial apoptotic pathway [11].

Normal functional disturbances in endoplasmic reticulum (ER) lead to ER stress. The ER stress pathway is considered as one of the three classic apoptosis signaling pathways and has been implicated in the development of several diseases. Our previous study demonstrated that kaempferol induces apoptosis in hepatocarcinoma cells via activation of ER stress pathway and the pro-apoptotic factor, C/EBP homologous protein (CHOP) [12]. CHOP is the point of convergence for the three-major upstream ER stress transducers and is also the best-characterized factor in the transition from ER stress to apoptosis [13–17]. In addition to apoptosis, cellular suicide can also be executed via non-apoptotic form of programmed cell death called autophagic cell death. Autophagy is a wide spread physiological process in eukaryotic cells at a basal level to assure cellular homeostasis [18]. Macroautophagy (hereafter called autophagy) is identified by the presence of double membrane organelle known as an autophagosome, which engulfs cytoplasmic components, including excessive, long-lived proteins or dysfunctional organelles, and subsequently deliver them to lysosomes for degradation. A variety of autophagy-related genes proteins, such as Atg5, Atg7, LC3, P62 and Beclin 1 are involved in the regulation of autophagy. For example, LC3 conversion from LC3-I to LC3-II is a critical determinant of autophagy, and the emergence of LC3-II marks the occurrence of autophagy [19–21]. Generally, autophagy plays a pro-survival role during stress response. However, the overactivation of autophagy contributes to autophagic cell death. In recent years, multiple hypotheses have been considered regarding the mechanisms of autophagy that are involved in cancer [22–24]. The current accepted hypothesis is that autophagy has dual and contradictory roles in carcinogenesis, but the precise mechanisms leading to autophagy in cancer are not yet fully defined. Huang et al reported the effect of autophagy in hepatocellular carcinoma, which demonstrated that kaempferol induces autophagic cell death in SK-HEP-1, a human hepatocellular carcinoma cell line [25].

Previous studies have demonstrated that several important signaling pathways mediate the complex cross-talk between apoptosis and autophagy, and the ER stress pathway can induce autophagy under certain circumstances [26]. Kouroku et al demonstrated that abnormal expression and accumulation of polyglutamine Q72 can induce ER stress, and then upregulate the expression levels of Atg12 and LC3-II to induce autophagy through PERK-EIf2a signaling pathway in mouse embryonal carcinoma cells [27]. Another study has reported that IREI-TRAF2-JNK is an important pathway in ER-stress-induced autophagy [28]. The present study aimed to explore the role of kaempferol in the apoptosis of hepatocarcinoma cells and the mechanisms.

## RESULTS

### Kaempferol triggers autophagy in a dose- and time-dependent manner

HepG2 cells and Huh 7 cells treated with Kaempferol increased the protein levels of Atg5, Atg7, Beclin1, and promoted a conversion from LC3-I to -II in a dose- and time-dependent manner, while decreased the protein level of Lamp1 (Figure 1B, 1D, Supplementary Figure 1A, 1B). The mRNA levels of Atg5, Atg7 and Beclin1 were also significantly increased and the mRNA levels of Lamp1 were significantly decreased (Figure 1C and 1E).

To evaluate the cytotoxicity of kaempferol, the viability of HepG2 cells incubated with 5∼100 μM kaempferol for 24 h was determined by using MTT assay (Supplementary Figure 2). Results showed that the value of 50% inhibitory concentration (IC50=20.37μM) and the viability of HepG2 cells was not influenced by treatment with <20μM kaempferol, but significant toxicities were observed with higher doses of kaempferol. In this study, we incubated the cells with 100μM of kaempferol for 24 h to explore the toxicities.

Autophagic flux was monitored after treatment with kaempferol in the presence or absence of chloroquine (CQ) in Huh 7 cells. As shown in Supplementary Figure 3, kaempferol treatment induced the lipidation of LC3I to LC3II and decreased the degradation of p62. The addition of CQ further increased LC3II conversion, and also further decreased the degradation of p62 compared with cells treated with kaempferol only. Taken together, the treatment of kaempferol directly inhibited autophagic flux in Huh 7 cells.

### Inhibition of autophagy alleviates kaempferol-induced HepG2 death

**Figure 2:**
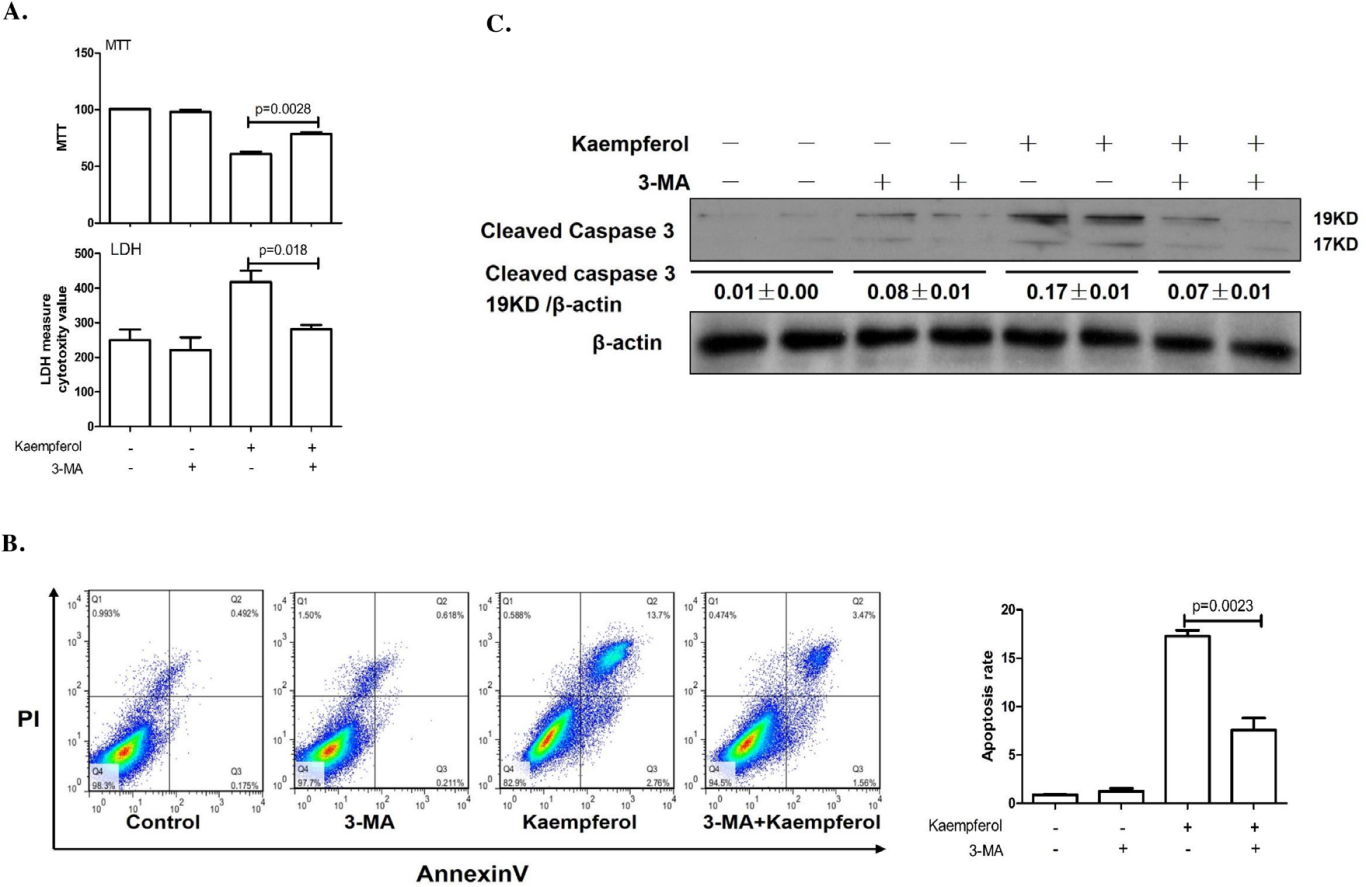
Autophagy inhibition by 3-MA alleviates kaempferol-induced death in HepG2 cells Data are expressed as mean ± SD of at least three independent experiments. **(A)** Cell viability was measured by MTT assay and cell death was measured by LDH activity analysis. **(B)** Apoptotic rate was determined using flow cytometry and quantified the apoptotic rate. **(C)** Protein expression levels of cleaved caspase3 and β-actin were determined using western blot and quantified by densitometry.

**Figure 3:**
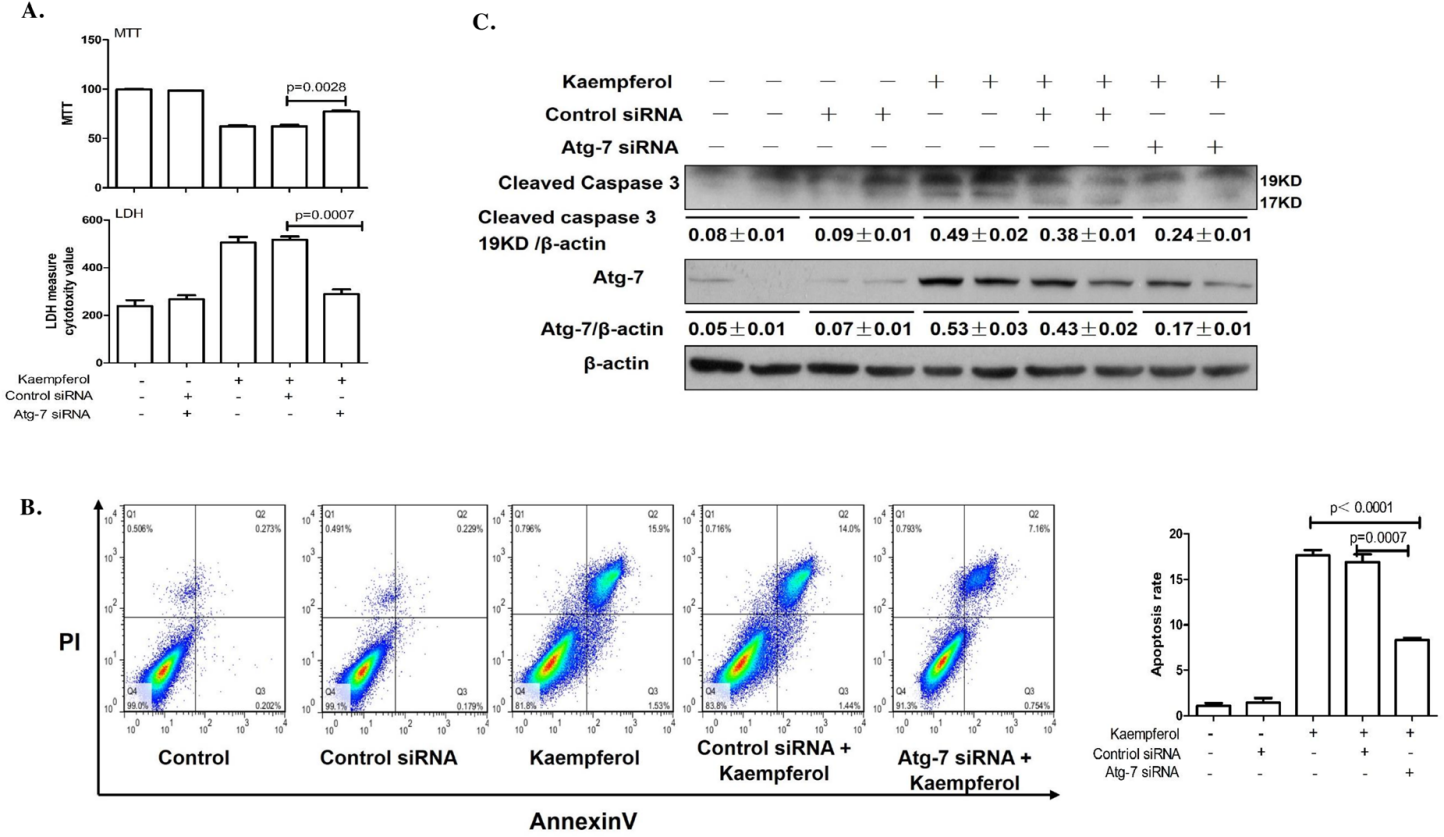
Knockdown of Atg7 with siRNA decreases kaempferol-induced death in HepG2 cells Data are expressed as mean ± SD of at least three independent experiments. **(A)** Cell viability was measured by MTT assay and cell death was measured by LDH activity analysis. **(B)** Apoptotic rate was determined using flow cytometry and quantified the apoptotic rate. **(C)** Protein expression levels of cleaved caspase3 and β-actin were determined using western blot and quantified by densitometry.

To explore the relationship between kaempferol-induced death and autophagy in HepG2 cells, the cells were pretreated with autophagy inhibitor, 3-MA to suppress autophagy or transfected with Atg7 siRNA to diminish the expression of Atg7. Compared with kaempferol only treatment group, cell viability was significantly increased in the 3-MA pretreatment group, and LDH activity and apoptotic rates were lower in the 3-MA pretreatment group compared with the kaempferol group (Figure 2A and 2B). Furthermore, the protein expression level of cleaved caspase3 was higher in the kaempferol treatment group, whereas it was reduced subsequently when autophagy was inhibited by 3-MA (Figure 2C). Following transfection with Atg7 siRNA, cell viability was significantly increased compared with the kaempferol treatment group. Additionally, LDH activity and the apoptotic rates were significantly reduced (Figure 3A and 3B). Western blot analysis also indicated that low expression of Atg7 suppressed the expression of cleaved caspase3 in kaempferol treatment group (Figure 3C). Inhibition of autophagy pathway, either through application of 3-MA or by knockdown of Atg7, strongly diminished the cell death. This indicated a pro-apoptotic function of autophagy. Based on our previous studies, the results indirectly proved that kaempferol can induce HCC cells death via autophagy pathway.

### Inhibition of ER stress reduces kaempferol-induced autophagy

**Figure 4:**
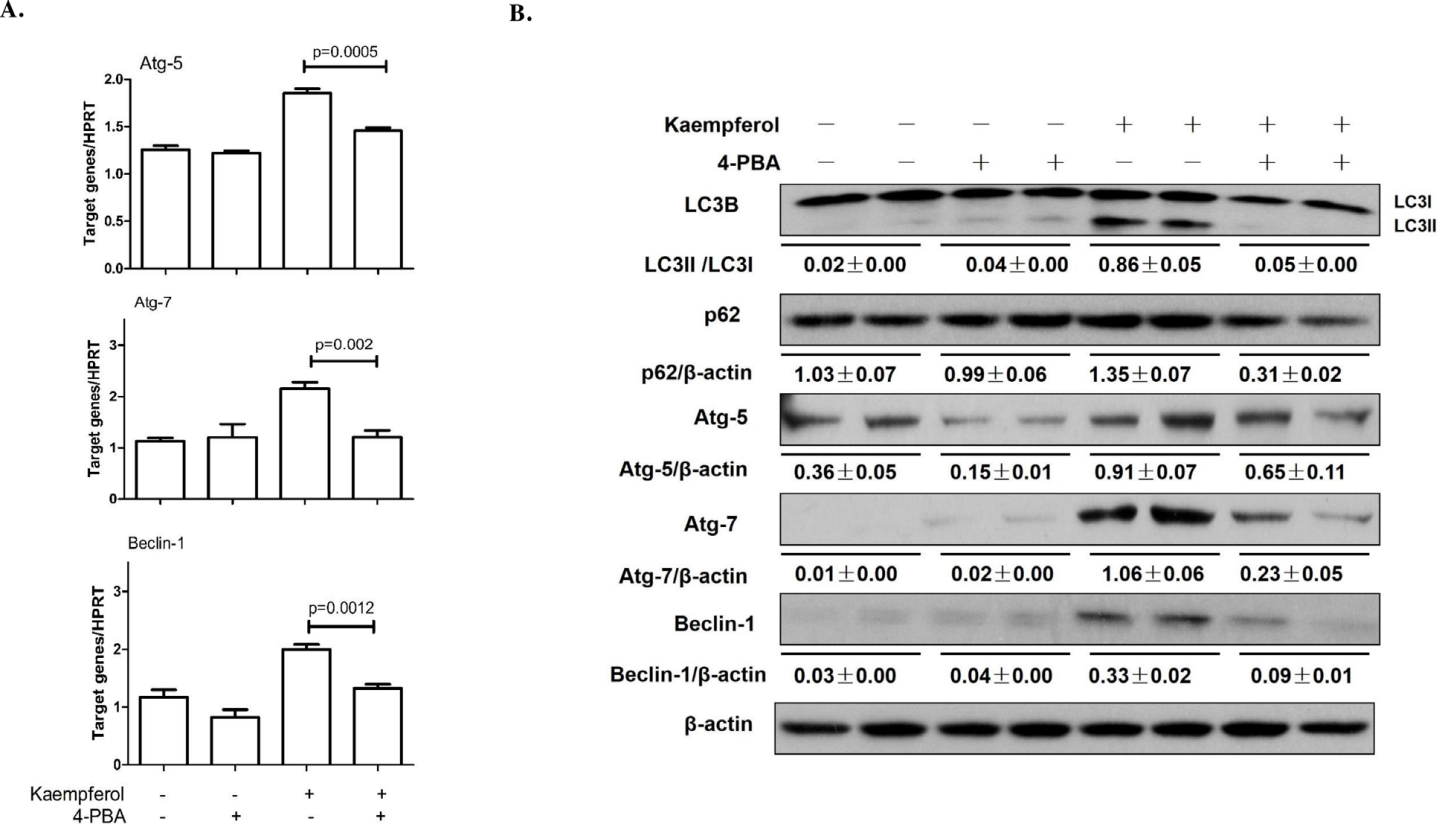
Endoplasmic reticulum stress inhibition by 4-PBA alleviates kaempferol-induced autophagy in HepG2 cells Data are expressed as mean ± SD of at least three independent experiments. **(A)** The mRNA expression levels of Atg5, Atg7 and Beclin1 were determined by qRT-PCR. **(B)** The protein expression levels of Atg5, Atg7, Beclin1, LC3B, p62and β-actin were determined by western blot and quantified by densitometry.

To confirm the role of the ER stress response in kaempferol-induced autophagy in HepG2 cells, we pretreated the cells with 4-PBA, an ER stress inhibitor. We found that 4-PBA suppressed Atg5, Atg7and Beclin1 gene expressions in both mRNA and protein levels and inhibited ER stress (Figure 4A). Moreover, 4-PBA prevented a conversion from LC3-I to LC3-II (Figure 4B). These results demonstrated that kaempferol-induced autophagy in HCC cells was associated with ER stress response.

### Kaempferol induces autophagy via ER stress-CHOP pathway in HepG2 cell

**Figure 5:**
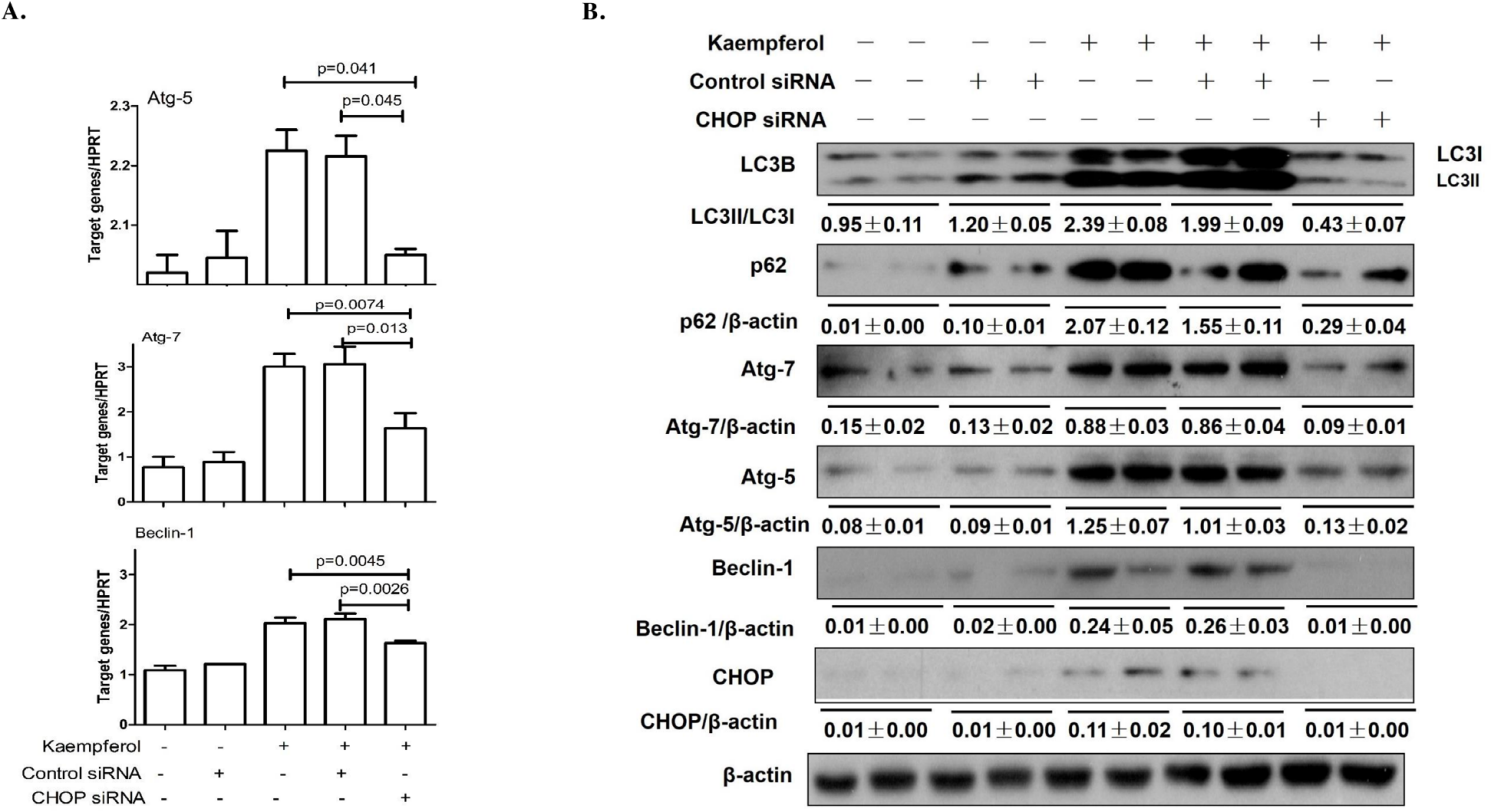
Knockdown of CHOP with siRNA remits kaempferol-induced autophagy Data are expressed as mean ± SD of at least three independent experiments. **(A)** The mRNA expression levels of Atg5, Atg7 and Beclin1 were determined by qRT-PCR. **(B)** The protein expression levels of Atg5, Atg7, Beclin1, LC3B, p62and β-actin were determined by western blot and quantified by densitometry.

**Figure 6:**
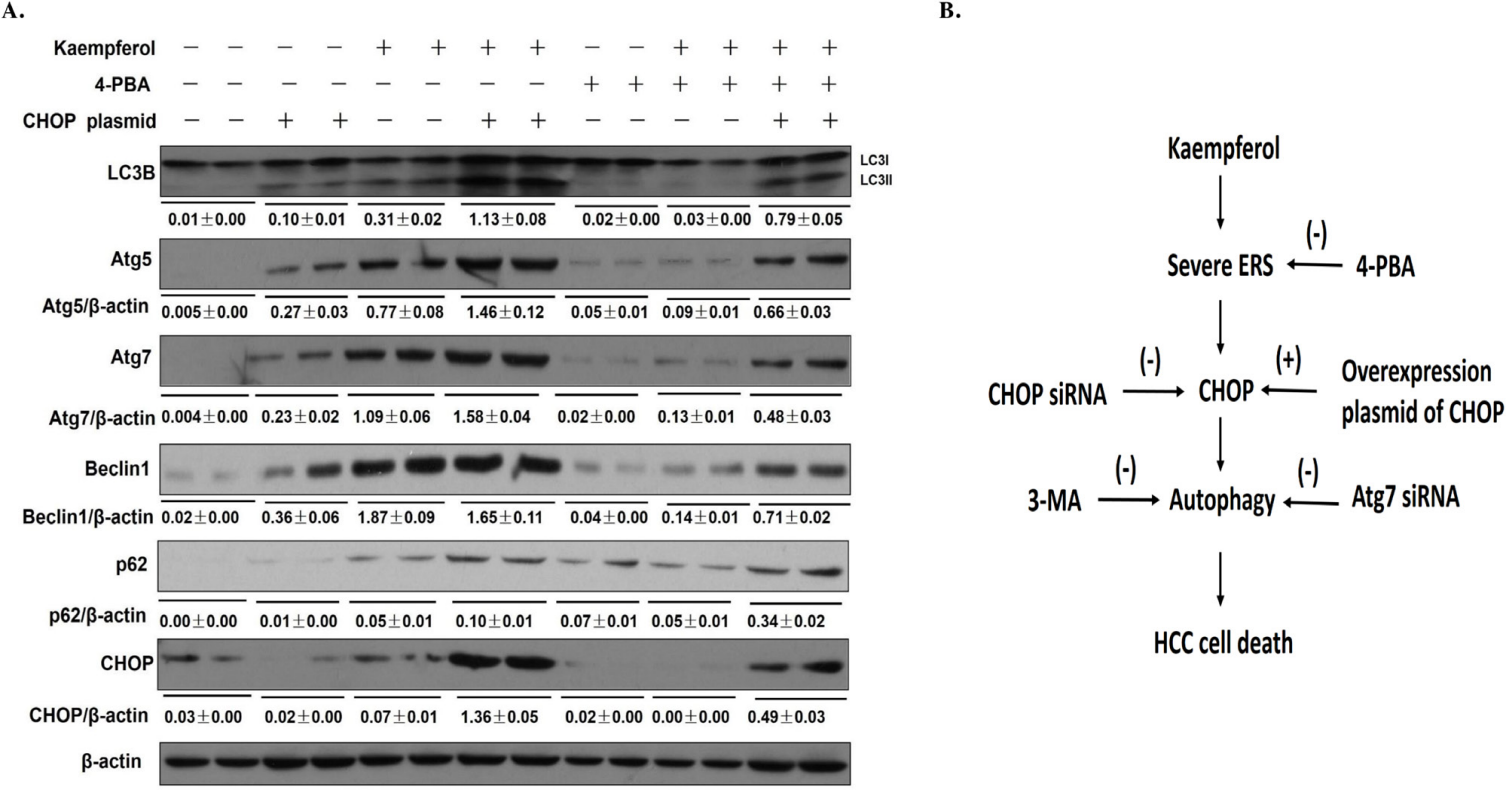
Transfection with CHOP-overexpression plasmid reverses the effect of 4-PBA on kaempferol-induced HepG2 cell autophagy Data are expressed as mean ± SD of at least three independent experiments. **(A)** The protein expression levels of Atg5, Atg7, Beclin1, LC3B, p62and β-actin were determined by western blot and quantified by densitometry. **(B)** In the kaempferol-induced HepG2 cells, kaempferol treatment induces severe ER stress, and then promotes the expression level of CHOP, which promotes autophagy to induce HCC cell death.

CHOP is a best-characterized factor in the transition from ER stress to apoptosis [30–31]. Our previous study demonstrated that CHOP plays a vital role in kaempferol-induced apoptosis via ER stress pathway [12]. Here, we investigated the function of CHOP in kaempferol-induced HepG2 autophagy by limiting its expression in HepG2 and Huh7 cells. Knockdown of CHOP with siRNA significantly attenuated the mRNA expression levels of Atg5, Atg7 and Beclin1 (Figure 5A), and decreased the protein expression levels of Atg5, Atg7, Beclin1 and LC3-II (Figure 5B, Supplementary Figure 4). Following transfection with CHOP-overexpression plasmid, the protein and mRNA expression levels of Atg5, Atg7, Beclin1 and LC3-II were markedly increased, resulting in the reversal of 4-PBA on kaempferol-induced cell autophagy (Figure 6A). These results indicated that high expression of CHOP promotes cell autophagy and kaempferol can trigger ER stress to induce autophagy via CHOP pathway.

## DISCUSSION

The current study indicated that kaempferol induces autophagy in HCC cells and to our knowledge, this is the first study to demonstrate that kaempferol triggers ER stress to promote cell death via CHOP-autophagy signaling pathway (Figure 6B).

Autophagy is a physiological process that occurs in normal cells at a basal level to assure cellular homeostasis [18]. In general, autophagy acts as a pro-survival stress response. However, there is also substantial evidence that over-activation of autophagy can lead to autophagic cell death in many cells [19–21]. In recent years, the dual and contradictory roles of autophagy presented in various cancer cells have been studied [22–24]. Therefore, study on autophagy of HCC cells is helpful to further understand the molecular mechanisms of the occurrence and development of hepatocellular carcinoma and subsequently guide the treatment. Our results indicated that kaempferol induces autophagy in HCC cells in a dose- and time-dependent manner which were in agreement with the previous study [25]. Moreover, autophagy inhibition by 3-MA protected HepG2 cells from kaempferol-induced death, and the protein expression of cleaved caspase3 was markedly decreased in the 3-MA pretreatment group. We also transfected HepG2 cells with Atg7 siRNA to suppress autophagy, and results demonstrated that inhibition of autophagy pathway, either through application of 3-MA or by knockdown of Atg7, strongly diminished cell death, indicating a pro-apoptotic function of autophagy. Based on our and other previous study results, we concluded that kaempferol-induced autophagy promotes ER stress-induced HepG2 cell death.

CHOP is the best-characterized factor in the transition from ER stress to apoptosis [30–31]. To examine the contribution of CHOP in kaempferol-induced cell death, we treated HepG2 cells with ER stress inhibitor, 4-PBA, or transfected HepG2 cells with CHOP siRNA and found that 4-PBA pretreatment or CHOP siRNA decreased the protein levels of Atg5, Atg7, Beclin1 and LC3-II, and attenuated kaempferol-induced autophagy. In addition, to confirm the role of CHOP in kaempferol-induced autophagy, we transfected HCC cells with a CHOP-overexpression plasmid and found that CHOP-overexpression significantly increased the expression of autophagy-related genes which increased kaempferol related autophagy. These data indicated that the expression level of CHOP impacts the occurrence of autophagy and also clarified that the role of CHOP in kaempferol-induced autophagy is via ER stress.

In conclusion, the present study results have extended the evidence to the previous studies and confirmed that kaempferol induces autophagy in hepatocellular carcinoma cells. Several important signaling pathways mediate the complex cross-talk between cell death and autophagy, and our results strongly suggested that CHOP plays an important role in kaempferol-induced autophagy. However, other additional mechanisms involved in kaempferol-induced autophagy requires further investigation before the application of kaempferol to HCC treatment.

## MATERIALS AND METHODS

### Cells culture and treatment

Human hepatoblastoma cell line (HepG2) and hepatocarcinoma cell line (Huh7) were purchased from the American Type Culture Collection (Manassas, VA, USA); kaempferol, dimethyl sulfoxide (DMSO), 4-Phenyl butyric acid (4-PBA) and other reagents not specified are from Sigma-Aldrich (St. Louis, MO, USA). The cells were maintained in Dulbecco’s Modified Eagle’s Medium (Hyclone; GE Healthcare Life Sciences, Logan, UT, USA) supplemented with 10% fetal bovine serum (Hyclone; GE Healthcare Life Sciences), antibiotics (100 U/ml penicillin and 100 mg/ml streptomycin; Beyotime Institute of Biotechnology, Haimen, China). Cells were incubated overnight at 37°C under a humidified atmosphere of 5% CO_2_ and then seeded at a density of 1 × 10^5^ cells/well in 12-well culture plates. Subsequently, the cells were incubated with different concentrations (0, 5, 10, 25, 50 and 100 μM) of kaempferol for 24 h. Other group of HepG2 cells was treated with 100 μM kaempferol for 0, 3, 6, 12 and 24h. Kaempferol was dissolved in DMSO (the final concentration of DMSO <0.1%). To determine how kaempferol triggers autophagy via ER stress-CHOP pathway, we did three additional experiments: i) 4-PBA pretreatment: cells were pretreated with 4-PBA at 1 mM for 30 min; ii) Interfering RNA (siRNA) Transfection: using siRNA to silence CHOP; and iii) CHOP overexpression plasmid (Shanghai Gene Pharma Co., Ltd., Shanghai, China) transfection: transfecting cells with CHOP-plasmid.

### Reverse transcription-quantitative polymerase chain reaction (RT-qPCR) analysis

The mRNA levels of target genes were evaluated using RT-qPCR. Total RNA was extracted from HepG2 cells using TRIzol reagent and then reverse-transcribed into cDNA by PrimeScript First Strand cDNA Synthesis Kit (TaKaRa Bio, Inc., Otsu, Japan) according to the manufacturer’s protocol. The hypoxanthine phosphoribosyl transferase (HPRT) gene was selected as an endogenous control. PCR was performed in a reaction mixture (20 μl) containing 4μl cDNA, 0.4 μl primer (10μM) of each, 5.2 μl diethylpyrocarbonate water and 10 μl SYBR Green (TaKaRa Bio, Inc.) using a quantitative PCR machine (ABI Prism 7500; Applied Biosystems Inc Waltham, MA, USA). Amplification conditions were: 50°C (2 min), 95°C (5 min) followed by 50 cycles of 95°C (15 s), 60°C (30 s). The mRNA levels were calculated using the 2^–ΔΔCt^ method [29]. The specific primer sequences for these genes were as follows: HPRT: 5’-TCAACGGGGGACATAAAAGT-3’ (forward), 5’-TGCATTGTTTTACCAGTGTCAA-3’ (reverse); Atg5: 5’-TGGGATTGCAAAATGACAGA-3’ (forward), 5’-TTCCCCATCTTCAGGATCAA-3’ (reverse); Atg7: 5’-ACCCAGAAGAAGCTGAACGA-3’ (forward), 5’-CTCATTTGCTGCTTGTTCCA-3’ (reverse);Beclin1: 5’-AGGTTGAGAAAGGCGAGACA-3’ (forward), 5’-AATTGTGAGGACACCCAAGC-3’(reverse).

### Western blot analysis

The cells were scraped off and washed with ice-cold phosphate-buffered saline (PBS), and then lysed with radioimmunoprecipitation assay buffer containing a mixture of protease inhibitors. A total of 30 μg protein from each sample was separated by 12 % sodium dodecyl sulfate-polyacrylamide gel electrophoresis at 80 v for 30 min and 120 v for 1 h, and then electrotransferred onto nitrocellulose membranes (Bio-Rad Laboratories, Inc. Hercules, CA, USA) using the Bio-Rad transfer blotting system. The membranes were subsequently incubated with 5% skim milk in Tris-buffered saline with Tween-20 for 1 h to block nonspecific binding and then incubated overnight with antibodies against Atg5, Atg7, Beclin1, LC3, cleaved caspase3, lamp1 and β-actin (Cell Signaling Technology, Inc., Danvers, MA, USA) at 4°C, followed by incubation with secondary antibody (Cell Signaling Technology, Inc.) for 1 h at room temperature. Proteins were visualized using enhanced chemiluminescence commercial kit (Thermo Fisher Scientific, Inc., Rockford, IL, USA).

### 3-(4,5-dimethylthiazol-2-yl)-2,5-diphenyltetrazolium bromide (MTT) assay for cell viability

In order to study the molecular mechanisms of kaempferol-induced autophagy, we inhibited the autophagy pathway through autophagy inhibitor 3-methyladenine (3-MA) at 1 mM for 1 h or by knockdown of Atg7 with siRNA. HepG2 cells were cultured in 96-well plates at a concentration of 1 × 10^4^ cells/well. After overnight growth, the cells were treated with 3-MA or transfection with Atg7 siRNA as described above, and then exposed to 100 μM kaempferol for 24 h. The vehicle (VE) and blank control (BC) groups were included. At the end of each treatment, 200 μl culture medium containing 0.5 mg/ml MTT (Amresco LLC, Solon, OH, USA) was added to each well and the mixture was incubated at 37°C for 4 h. The supernatant was removed, crystals were dissolved in 150μl DMSO, and the absorbance was measured at 570 nm using a microplate reader (Multiskan MK3; Bio-RadLaboratories, Inc.). Cell viability was calculated as: [(A_kaempferol treatment group_– A_BC_)/(A_VE_–A_BC_)] × 100%, (A represents the absorbance).

### Lactate dehydrogenase (LDH) activity assay

The colorimetric LDH activity assay kit (Applygen Technologies, Inc., Beijing, China) was used to quantify the amount of LDH released into the supernatant from the damaged cells. HepG2 cells were incubated in 96-well culture plates, and then treated with 3-MA or transfected with Atg7 siRNA as described above. The supernatants from each treatment group were collected to perform the LDH activity assay according to the manufacturer’s protocol.

### Flow cytometric analysis

The AnnexinV–fluorescein isothiocyanate (FITC)/propidium iodide (PI) double-staining assay (Nanjing Key Gen Biotech, Co., Nanjing, China) was used to quantify cell apoptosis. HepG2 cells were cultivated in 60-mm culture plates for 24 h at 37°C. Following exposure to the indicated treatments, cells were harvested by trypsinization, washed with PBS, pelleted by centrifugation at 800 × g at 4°C for 5 min, and resuspended in 0.5 ml binding buffer (Nanjing KeyGen Biotech, Co., Ltd. Nanjing, China). The cells were incubated with 5 μl Annexin V–FITC and 5 μl PI working solution for 15 min at room temperature in dark. The samples were analyzed on a FACScan flow cytometer (BD Biosciences, Franklin Lakes, NJ, USA) using FlowJo software (version 7.6; FlowJo LLC, Ashland, OR, USA). Double staining of cells with Annexin V–FITC and PI enabled the identification of different cell populations based on their staining patterns, which were as follows: lower left quadrant, live cells (FITC–PI–); lower right quadrant, early apoptotic cells (FITC+PI–); upper right quadrant, late apoptotic cells (FITC+PI+); upper left quadrant, necrotic cells (FITC–PI+).

### Small interfering RNA (siRNA) treatment

At 24h before transfection, HepG2 cells were prepared in 12-well culture plates. Cells were transfected with 20μM human Atg7 siRNA and negative control siRNA using Lipofectamine 2000 reagent for 6 h according to the manufacturer’s protocol (Shanghai GenePharma Co., Ltd.). Atg7 siRNA sequences were designed as follows: Forward (F) 5’- CAGCTCTGAACTCAATAATAA-3’. The transfected cells were then treated further with 100μM kaempferol for 24 h further. The transfection procedure of CHOP siRNA is the same as Atg7 siRNA, and the CHOP siRNA sequences were designed as follows: Forward (F) 5’-GAGCUCUGAUUGACCGAAUTT-3’.

### CHOP overexpression plasmid treatment

HepG2 cells were cultured in 12-well culture plates of 1ml volumes. CHOP overexpression plasmid and the empty vector control plasmid were transfected into HepG2 cells using Lipofectamine 2000 reagent according to the manufacturer’s protocol. Following a 24 h transfection, HepG2 cells were treated with ER stress inhibitor, 4-PBA at 1 mM for 30 min, and cultured with 100 μM kaempferol for an additional 24h.

### Statistical analysis

Data were expressed as mean ± SD from at least three separate experiments. Statistical comparisons between the groups were performed using Student’s t-test. Differences were considered statistically significant at P<0.05.

## SUPPLEMENTARY MATERIALS FIGURES


