# Supplementary material for: Kaempferol induces hepatocellular carcinoma cell death via endoplasmic reticulum stress-CHOP-autophagy signaling pathway
Source: Oncotarget. 2017 Jul 12;8(47):82207–16. doi: 10.18632/oncotarget.19200 (PMC5669883; doi:10.18632/oncotarget.19200)
Supplement: Supplementary file 1 [file oncotarget-08-82207-s001.pdf]

Kaempferol induces hepatocellular carcinoma cell death via endoplasmic reticulum stress-CHOP-autophagy signaling pathway

SUPPLEMENTARY MATERIALS

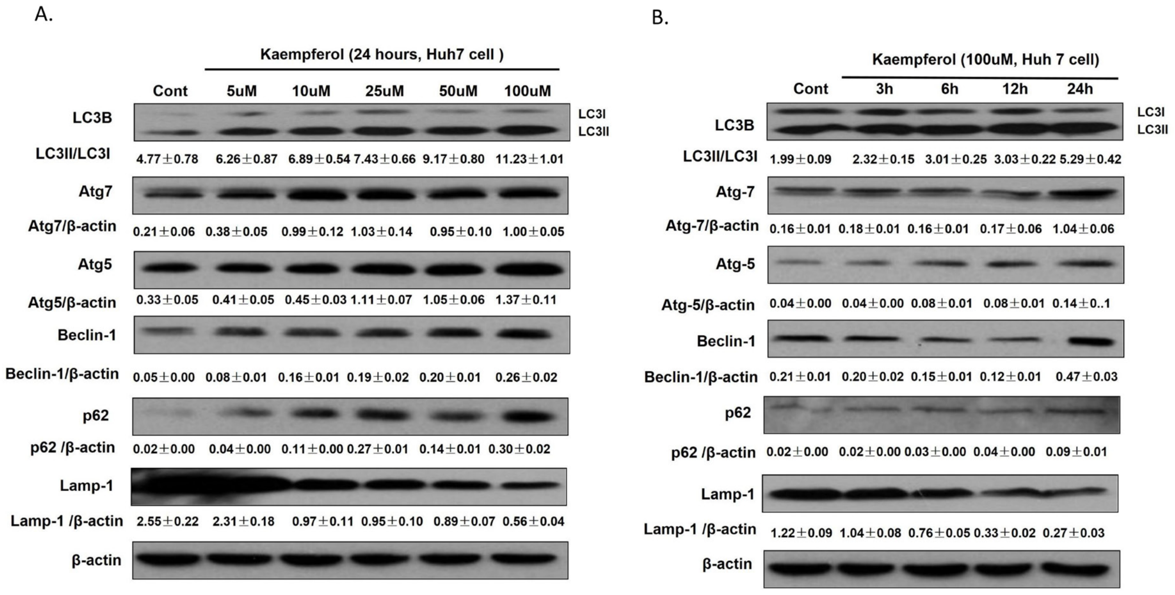

Supplementary Figure 1: Dose- and time-dependent effects of kaempferol on protein of autophagy-related genes in Huh 7 cells. Data are expressed as mean ± SD of at least three independent experiments. **(A)** For dose-dependent effects, Huh 7 cells were treated in the presence or absence of different concentrations of kaempferol for 24 h. The protein expression levels of Atg5, Atg7, Beclin1, Lamp1, p62, LC3B and β-actin were measured by western blot. Data are expressed as the mean ± SD of at least three independent experiments. **(B)** For time-dependent effects, Huh 7 cells were exposed to 100 μM kaempferol for different time periods. The protein expression levels of Atg5, Atg7, Beclin1, Lamp1, p62, LC3B and β-actin were measured by western blot. Data are expressed as the mean ± SD of at least three independent experiments.

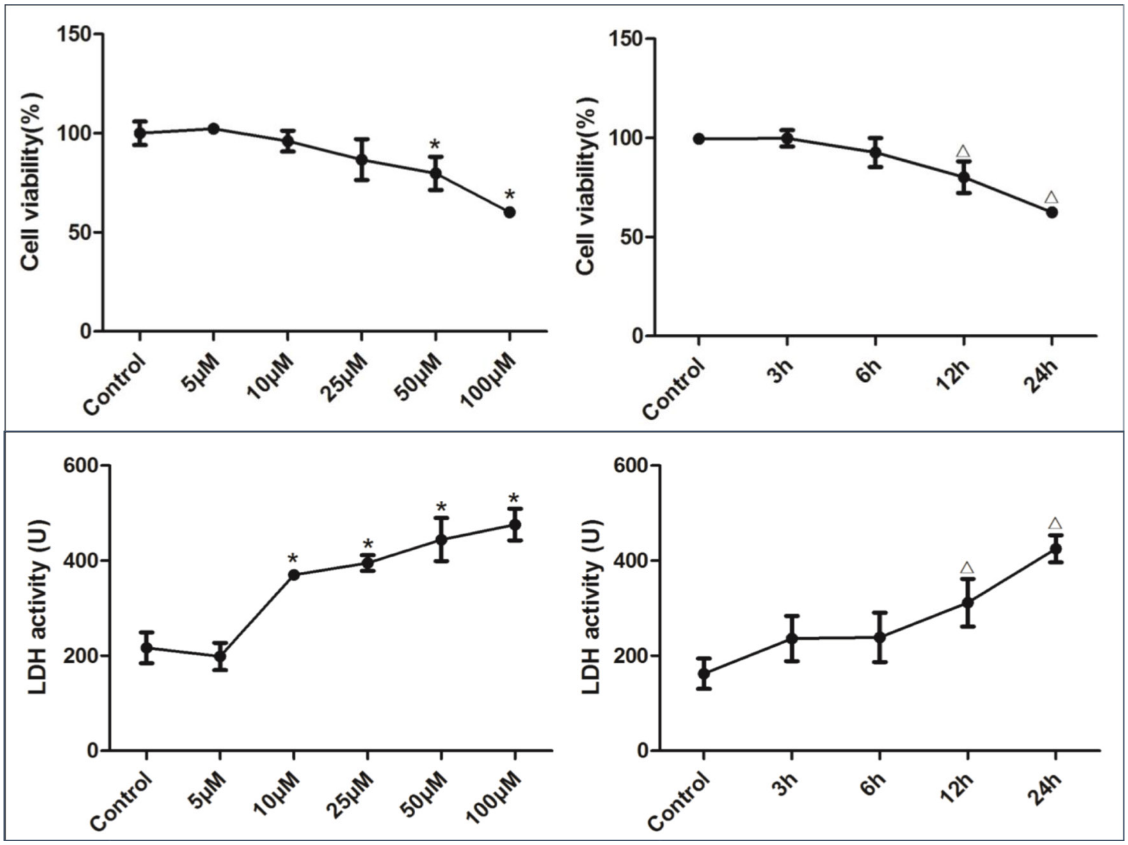

Supplementary Figure 2: Dose- and time-dependent effects of kaempferol on HepG2 cells. Data are expressed as the mean ± SD of at least three independent experiments. HepG2 cells were treated with 5, 10, 25, 50 and 100 μM kaempferol for 24 h, and Hep G2 cells were treated with 100 μM kaempferol for 0, 3, 6, 12 and 24 h. All data are expressed as mean ± SD of at least three independent experiments. Cell viability was evaluated using MTT assay and cell death was evaluated by measuring LDH activity in the supernatant.

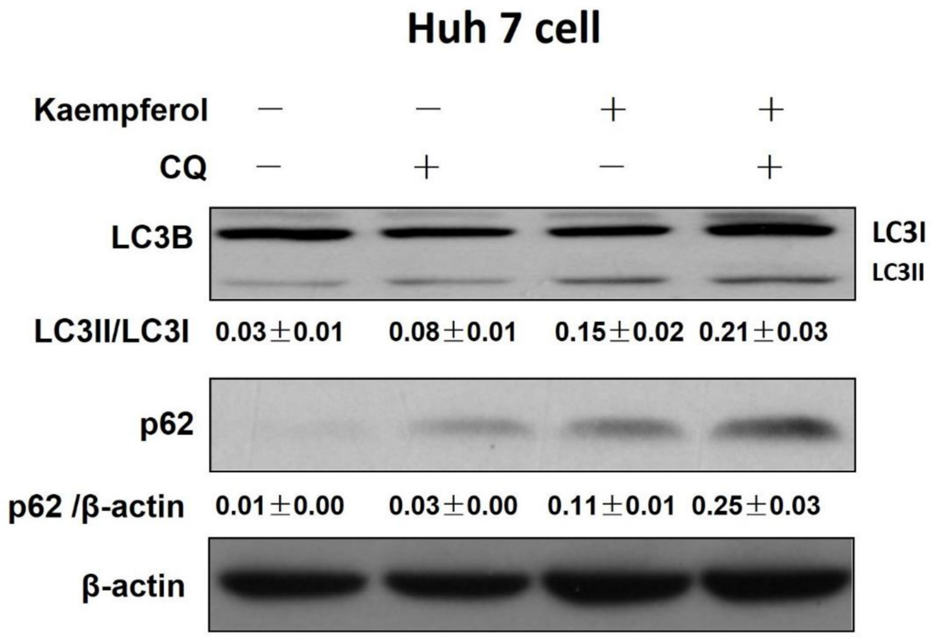

Supplementary Figure 3: Kaempferol treatment inhibits autophagic flux in the Huh7 cells. Data are expressed as the mean ± SD of at least three independent experiments. Huh 7 were treated with 100 μM kaempferol in the absence or presence of CQ (10 μM) for 24 hours. Western blot showing the LC3BII/I ratio and p62 degradation. β-actin is shown as a loading control.

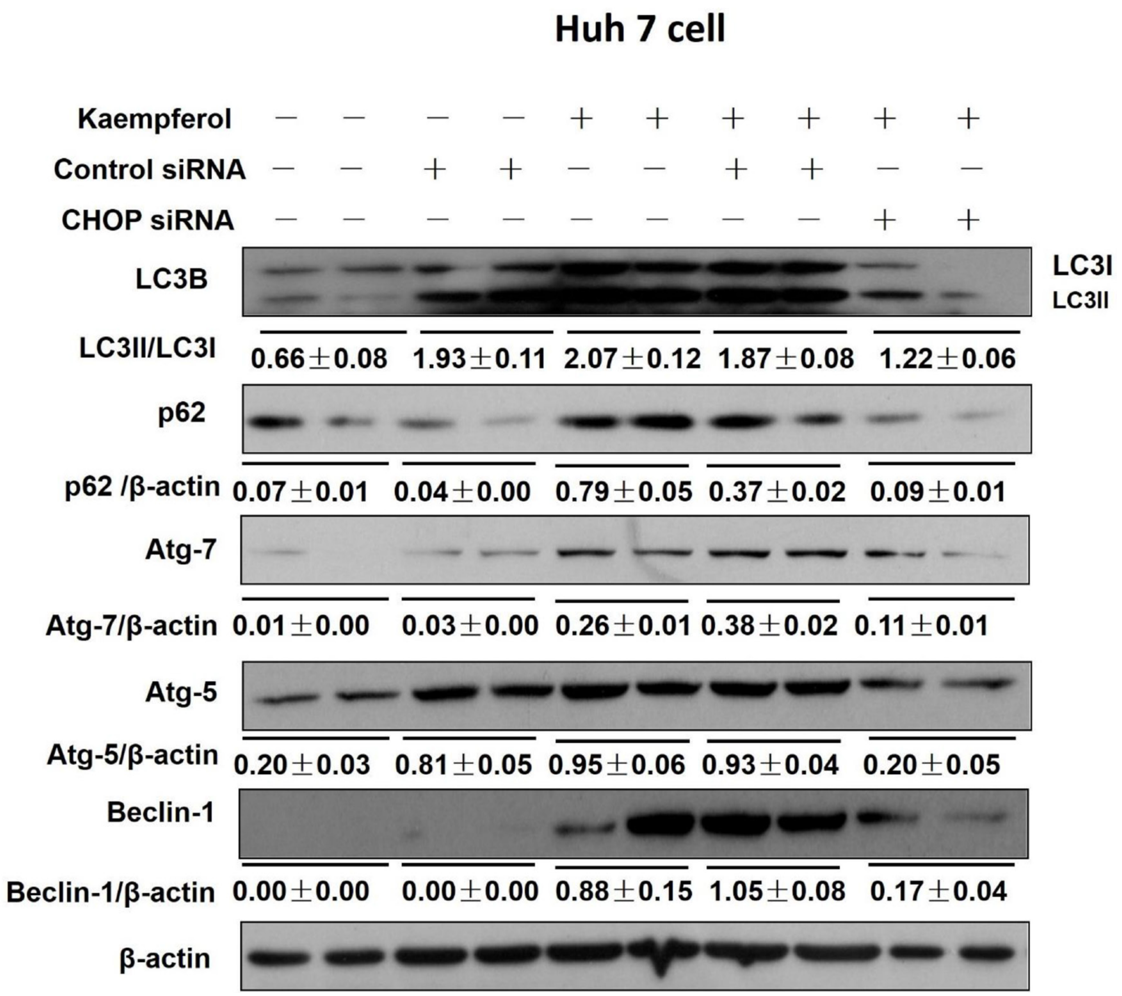

Supplementary Figure 4: Knockdown of CHOP with siRNA remits kaempferol-induced autophagy in Huh 7 cells. Data are expressed as the mean ± SD of at least three independent experiments. The protein expression levels of Atg5, Atg7, Beclin1, LC3B, p62 and β-actin were determined by western blot and quantified by densitometry.
